# A Conserved Phenylalanine Residue of Autographa Californica Multiple Nucleopolyhedrovirus AC75 Protein Is Required for Occlusion Body Formation

**DOI:** 10.3389/fmicb.2021.663506

**Published:** 2021-04-08

**Authors:** Xingang Chen, Jian Yang, Xiaoqin Yang, Chengfeng Lei, Xiulian Sun, Jia Hu

**Affiliations:** ^1^Wuhan Institute of Virology, Center for Biosafety Mega-Science, Chinese Academy of Sciences, Wuhan, China; ^2^College of Life Sciences, University of Chinese Academy of Sciences, Beijing, China

**Keywords:** Autographa californica multiple nucleopolyhedrovirus, AC75, Phe-54, budded virus, occlusion body

## Abstract

Autographa californica multiple nucleopolyhedrovirus (AcMNPV) *orf75* (*ac75*) is a highly conserved gene that is essential for AcMNPV propagation. However, the key domains or residues of the AC75 protein that play a role in viral propagation have not been identified. In this study, sequence alignment revealed that residues Phe-54 and Gln-81 of AC75 were highly conserved among alphabaculoviruses and betabaculoviurses. Thus, Phe-54 and Gln-81 AC75 mutation bacmids were constructed. We found that Gln-81 was not required for viral propagation, whereas mutating Phe-54 reduced budded virus production by 10-fold and impaired occlusion body formation when compared with that of the wild-type AcMNPV. Electron microscopy observations showed that the Phe-54 mutation affected polyhedrin assembly and also occlusion-derived virus embedding, whereas western blot analysis revealed that mutating Phe-54 reduced the amount of AC75 but did not affect the localization of AC75 in infected cells. A protein stability assay showed that the Phe-54 mutation affected AC75 stability. Taken together, Phe-54 was identified as an important residue of AC75, and *ac75* is a pivotal gene in budding virus production and occlusion body formation.

## Introduction

Autographa californica multiple nucleopolyhedrovirus (AcMNPV), the type species of the genus *Alphabaculovirus* in the family of *Baculoviridae*, has a circular, double-stranded DNA genome of 134 kb that codes for 154 genes (Ayres et al., 1994). AcMNPV produces two viral forms: budded viruses (BVs) and occlusion-derived viruses (ODVs) (van Oers and Vlak, 2007; Rohrmann, 2019). BVs are responsible for spreading AcMNPV infection among susceptible insect cells and tissues (Blissard and Wenz, 1992), whereas ODVs initiate primary infection in the midgut epithelium of insects (Braunagel and Summers, 2007; Hodgson et al., 2007). AcMNPV genome replication and nucleocapsid assembly both occur in the nucleus. The synthesized nucleocapsids egress from the nucleus and bud from the plasma membrane to form mature BVs, whereas the retained nucleocapsids are enveloped with intranuclear microvesicles to form ODVs that are further enclosed within polyhedrins to form occlusion bodies (OBs).

Baculovirus genome replication and transcription occur in the virogenic stroma (VS) (Young et al., 1993). The baculovirus genome is packaged into pre-assembled capsid sheaths to form mature nucleocapsids. Subsequently, a subset of the nucleocapsids egress from the nucleus to produce BVs. According to a previous report, AcMNPV nucleocapsid egress involves actin-based movement within nuclear envelope protrusions, which is coupled with localized nuclear envelope disruption and viral release into the cytoplasm (Ohkawa and Welch, 2018). Currently, several AcMNPV genes (*ac11*, *ac13*, *ac51*, *ac66*, *ac78, gp41*, *ac93*, *p48*, *exon0*, and *p49*) have been identified to be required for nuclear egress (Fang et al., 2007; Ke et al., 2008; McCarthy et al., 2008; Yuan et al., 2008, 2011; Tao et al., 2013, 2015; Biswas et al., 2017; Li et al., 2018; Qiu et al., 2019; Chen et al., 2020). Deletion of these genes does not affect viral genome replication and progeny nucleocapsid assembly but restrains or blocks the egress of progeny nucleocapsids from the nucleus to the cytoplasm. Recent research has shown that a group of AcMNPV proteins interact with components of the endosomal sorting complex required for transport-III complex and this complex is required for nuclear egress (Yue et al., 2018).

Nucleocapsids retained in nuclei are enveloped by intranuclear microvesicles to form ODVs and are further enclosed within the polyhedrins to form OBs (Rohrmann, 2019). The assembly and occlusion of ODVs, including intranuclear microvesicle formation, involves nucleocapsid bundles adhering to microvesicles, and subsequent wrapping of nucleocapsids by these microvesicles (Blissard and Theilmann, 2018). In previous reports, a number of AcMNPV genes (*ac11*, *ac76*, *ac93*, *odv-e25*, *p48*, and *p49*) have been shown to be involved in ODV envelopment (McCarthy et al., 2008; Yuan et al., 2008, 2011; Hu et al., 2010; Chen et al., 2012; Tao et al., 2015). Among the aforementioned genes, three genes (*ac11*, *ac93*, and *p48*) are required for both nuclear egress of the nucleocapsids and formation of intranuclear microvesicles (McCarthy et al., 2008; Yuan et al., 2008, 2011; Tao et al., 2015; Wang et al., 2019). The OBs form after the mature ODVs are embedded within polyhedrins. Then, a carbohydrate-composed outer layer and the polyhedral envelope protein (PEP) are supplemented to the surface of mature OBs (Whitt and Manning, 1988; Russell and Rohrmann, 1990), where a functional P10 protein associated with nuclear fibrillar structures is also required (Williams et al., 1989; Carpentier et al., 2008). In deletion mutants of *pep* or *p10*, OBs are irregular and fragile and no outer calyx layer is observed (Vlak et al., 1988; Li et al., 2015). Although several genes essential for OB morphogenesis have been reported, the detailed mechanism of OB morphogenesis is poorly understood.

*ac75* is a highly conserved gene found in all sequenced baculovirus genomes, except Culex nigripalpus nucleopolyhedrovirus (CuniNPV), and *ac75* is predicted to encode a protein of 133 amino acids in length with a putative molecular mass of 15.5 kDa. AC75 localizes predominantly in the intranuclear ring zone in the late infection phase, while also exhibiting a nuclear rim distribution during the early phase (Guo et al., 2017; Shi et al., 2017). AC75 has been reported to be associated with the envelope and nucleocapsid fractions of BVs but only with the nucleocapsid fraction of ODVs (Shi et al., 2017). In contrast, another report revealed that AC75 is associated with the nucleocapsid of BVs and with both the envelope and nucleocapsid of ODVs (Guo et al., 2017). Nevertheless, two reports both confirmed that *ac75* was required for nucleocapsid egress and intranuclear microvesicle formation (Guo et al., 2017; Shi et al., 2017).

In this study, sequence alignment revealed that Phe-54 and Gln-81 of AC75 were highly conserved among alphabaculoviruses and betabaculoviurses. We constructed Phe-54 and Gln-81 point mutation bacmids (bAc*^*ac*75*F*54*S*^*-*ph* and bAc*^*ac*75*Q*81*A*^*-*ph*) and evaluated the effects of these mutations on virus proliferation. The data showed that the Gln-81 mutation did not affect viral propagation, whereas the Phe-54 mutation impaired BV production, polyhedrin assembly and ODV embedding. Thus, the results showed that Phe-54 is a key residue of AC75, and *ac75* is essential for OB morphogenesis.

## Materials and Methods

### Cell Lines, Viruses, Insects, and Antibodies

Sf9 cells (Invitrogen, Carlsbad, CA, United States) were cultured at 27°C in Grace’s insect medium (Invitrogen) supplemented with 10% (v/v) fetal bovine serum (Gibco, Grand Island, NY, United States) and 0.1% (v/v) antibiotic-antimycotic solution (Invitrogen). The recombinant bacmids were generated from bMON14272 (Invitrogen) and maintained in *Escherichia coli* (*E. coli*) strain DH10B (Invitrogen), which also contained helper plasmids for homologous recombination and transposition. *Spodoptera exigua* (*S. exigua*) larvae were reared on an artificial diet at 28°C (Shorey and Hale, 1965).

The polyclonal antiserum of anti-AC75 was prepared in rabbits according to previously published methods (Li et al., 2018). The anti-POLH polyclonal antiserum to detect the polyhedrin protein was preserved in the laboratory. Mouse monoclonal anti-actin antibody, horseradish peroxidase (HRP)-conjugated goat anti-mouse antibody and HRP-conjugated goat anti-rabbit antibody were purchased from Proteintech (Wuhan, China).

### Construction of an *ac75* Knockout Bacmid

The AcMNPV *ac75* gene was deleted using the λ red recombination system in *E. coli* BW25113 cells (containing bMON14727 and pKD46) as described previously (Datsenko and Wanner, 2000; Li et al., 2015). The *ac75*-null bacmid was constructed by replacing a 112-bp fragment of the *ac75* ORF with a chloramphenicol resistance gene (*CmR*) cassette and retaining 115 nt of the 5′-end and 175 nt of the 3′-end of the *ac75* ORF to avoid affecting transcription of neighboring genes *ac76* and *ac74.* The *ac75* knockout bacmid was verified by PCR (primer sequences are shown in Table 1) and termed bAc*^*ac*75^*^KO^.

**TABLE 1:** Primers used in this study.

Primer name	Primer sequence (5′-3′)^a^
*ac75*-US-F (*Sac*I)	CGAGCTCCGCAACGAATAGAGTAAGGG
*ac75*-US-R (*Bam*HI)	CGGGATCCGATAGACTTGTTCGCACAGC
*CmR*-F (*Bam*HI)	CGGGATCCTGTAGGCTGGAGCTGC
*CmR* -R (*Hin*dIII)	CCCAAGCTTCATATGAATATCCTCCTTAGTTCC
*ac75*-DS-F (*Hin*dIII)	CCCAAGCTTACTCAGTAGGCGACAGGTTG
*ac75*-DS-R (*Xho*I)	CCGCTCGAGCTTTGGCGTGGTCAATG
*ph*-F (*Eco*RI)	CGGAATTCACCATCTCGCAAATAAATAAG
*ph*-R (*Sac*I)	CGAGCTCTGTATCGTGTTTTAATACGCC
*egfp*-F (*Sma*I)	CCCCGGGATGGTGAGCAAGGGCGAGGAGC
*egfp*-R (*Xho*I)	CCGCTCGAGTCACTTGTACAGCTCGTCCATGCCGAG
Dual-*ac75*-F1	CCGGAGTAGCCATATTTAGCCTAGTGTATGAC
Dual-*ac75*-R1	CAGAATTCTTAATACGCTGGCAGTTGGTATG
Dual*-ac75*-R2	TTACTTATCGTCGTCATCCTTGTAATCATACGCTGGCAG TTGGTATG
pFast-*ac75*-F1	CGTATTAAGAATTCTGCAGATATCCAGCAC
pFast-*ac75*-F2	TGACGACGATAAGTAAGAATTCTGCAGATATCCAGCAC
pFast-*ac75*-R1	AATATGGCTACTCCGGAATATTAATAGATCATGGAG
*ac75*^F54S^-F	AACTCAAACGAGTAGTTAACATGAGTTTAAACAATG
*ac75*^F54S^-R	TGTTTAAACTCATGTTAACTACTCGTTTGAGTTTAAGC
*ac75*^Q81A^-F	AGTAGGCGAGCGGTTGATTTTTTAATACATG
*ac75*^Q81A^-R	AAATCAACCGCTCGCCTACTGAGTTTATTAG
*ac75*-F	ATGTCCAATTTAATGAAAAACTTTTTCACC
*ac75*-R	TTAATACGCTGGCAGTTGGTATGC
*qie1*-F	TGTGATAAACAACCCAACGAC
*qie1*-R	GTTAACGAGTTGACGCTTG
*qpe38*-F	AATGGAACAGCAGCGAATGA
*qpe38*-R	GTCGCACGTAGTCGGAATC
*qgp64*-F	ACGACCTGATAGTCTCCGTG
*qgp64*-R	TGTAGCAATTACTGGTGTGTGC
*qvp39*-F	TTGCGCAACGACTTTATACC
*qvp39*-R	TAGACGGCTATTCCTCCACC
*qpolh*-F	TTAGGTGCCGTTATCAAGA
*qpolh*-R	GCCACTAGGTAGTTGTCT
*q18S*-F	TACCGATTGAATGATTTAGTGAGG
*q18S*-R	TACGGAAACCTTGTTACGACTTT
pIB-F1	GTCCAGTGTGGTGGAATTCTG
pIB-F2	CGGCGGCAGCGGCGGCGGCAGCCCCGGGATGGTG AGCAAGGGCGAGGAGC
pIB-R	TAGTGGATCCGAGCTCGGTAC
pIB-*egfp*-F	GAGCTCGGATCCACTAATGGTGAGCAAGG GCGAGGAGC
pIB-*egfp*-R	TTCCACCACACTGGACCTACTTGTACAGCTCGTC CATGCCGAG
pIB-*ac75*-F	GAGCTCGGATCCACTAATGTCCAATTTAATGAAAAA CTTTTTCACC
pIB-*ac75*-R	CGCCGCTGCCGCCGCCATACGCTGGCAGTTGGTATGC

*^*a*^Restriction sites and homologous sequences were underlined.*

### Construction of *ac75* Recombinant Bacmids

The AcMNPV *ac75* gene knockout and repair bacmids were generated as described previously (Chen et al., 2020). Briefly, the *polh* and *egfp* genes were, respectively, cloned downstream of the *polh* and *p10* gene promoters in pFastBacDual to generate donor plasmid pFBD-*ph*-*egfp*. The fragment containing the *ac75* promoter and ORF was inserted into pFBD-*ph*-*egfp* to give the recombinant plasmid pFBD-*ph*-*ac75*-*egfp*. Subsequently, the donor plasmids pFBD-*ph*-*egfp* and pFBD-*ph*-*ac75*-*egfp* were transformed into *E. coli* DH10B competent cells (containing the bacmid bAc*^*ac*75^*^KO^ and a helper plasmid). Recombinant bacmids were selected by gentamicin and kanamycin resistance with blue-white screening and further identified by PCR (primer sequences are shown in Table 1).

The *ac75* point mutation donor plasmids were constructed using FastCloning (Li et al., 2011) and the corresponding recombinant bacmids were generated via the bac-to-bac system. For example, to generate the AC75-F54S mutant donor plasmid, the fragment was amplified by using the primer pairs *ac75*^F54*S*^-F/R (sequences are shown in Table 1) and the template pFBD-*ph*-*ac75*-*egfp*. Then, 1 μL of the enzyme *Dpn*I (Takara, Toyoko, Japan) was added to the PCR product (9 μL) and digested at 37°C for 1 h. The mixture was transformed into *E. coli* DH5α competent cells. The recombinant plasmid selected by gentamicin and ampicillin resistance was further validated by PCR and DNA sequencing. The donor plasmid was termed pFBD-*ph*-*ac75*F54S-*egfp*. Subsequently, the mutation plasmid pFBD-*ph*-*ac75*F54S-*egfp* was further transformed into DH10B competent cells (containing the bAc*^*ac*75^*^KO^ bacmid and a helper plasmid) to generate mutation bacmid bAc*^*ac*75*F*54*S*^*-*ph* via the bac-to-bac system. The point mutation recombinant bacmid of bAc*^*ac*75*Q*81*A*^*-*ph* was also constructed by the same methods.

### Transmission Electron Microscopy and Immunoelectron Microscopy Analyses of Virus Infected Cells

Transmission electron microscopy (TEM) analysis was performed according to previous results (Qin et al., 2019). Sf9 cells infected with vAc-*ph*, vAc*^*ac*75*F*54*S*^*-*ph*, or vAc*^*ac*75^*^REP^-*ph* at an MOI of 5 were fixed with 2.5% (v/v) glutaraldehyde for 2 h and harvested at 24, 36, and 48 h post infection (p.i.). Ultrathin sections were visualized by a FEI Tecnai G^2^ 20 TWIN TEM. For immunoelectron microscopy analysis, Sf9 cells were infected with vAc-*ph*, vAc*^*ac*75*F*54*S*^*-*ph* or vAc*^*ac*75^*^REP^-*ph* at an MOI of 5. At 48 h p.i., the cells were fixed with 1% paraformaldehyde-0.5% glutaraldehyde for 10 min at 4°C, refixed with 2% paraformaldehyde-2.5% glutaraldehyde for 1 h at 4°C and then dehydrated and embedded according to previous methods (Xu et al., 2019). Ultrathin sections were immunostained with anti-AC75 pAb (1:50) as the primary antibody. Goat anti-rabbit IgG coated with gold particles (10 nm; Sigma, Darmstadt, Germany) was used as the secondary antibody (1:50). Ultrathin sections were also visualized by using the FEI Tecnai G^2^ 20 TWIN TEM.

### Scanning Electron Microscopy (SEM), TEM, and Negative Staining Analyses of OBs

OBs were amplified and isolated from the infected larvae according to the method described by Gross et al. (Gross et al., 1994). For SEM, OBs (10^8^ OBs/mL) were loaded onto the silver paper and dried at 37°C overnight. The samples were sputter coated with gold and examined by SEM (Hitachi SU8010). For TEM, OBs were fixed with 2.5% (v/v) glutaraldehyde for 2 h and prepared as described previously (Ji et al., 2015). Ultrathin section images were obtained by TEM (FEI Tecnai G^2^ 20 TWIN). For negative staining analysis, 10 μL of an OB suspension (10^8^ OBs/mL) was loaded onto a copper grid for 10 min. Filter paper was used to remove the remaining solution from the grid. Then, 10 μL dissolution buffer was added to dissolve the OBs for 1 min. After removing the dissolution buffer, the grid was stained with 2% (w/v) phosphotungstic acid (pH 5.7) for 1 min. The grid was also examined by TEM. The number of OBs with ODVs in a field of view were counted using ImageJ software^1^, and the numbers were analyzed using the Kruskal-Wallis test followed by Dunn’s multiple comparison test.

### Immunofluorescence Microscopy

To investigate whether AC75-F54S affected the localization of AC75, immunofluorescence assays were performed as described previously with minor modifications (Hepp et al., 2018). Briefly, Sf9 cells were seeded (4 × 10^5^ cells/dish) on a glass dish and allowed to attach for 2 h. The cells were infected with vAc-*ph*, vAc*^*ac*75*F*54*S*^*-*ph* or vAc*^*ac*75^*^REP^-*ph* at an MOI of five and fixed with 4% paraformaldehyde for 10 min at 15, 24, and 48 h p.i. After treatment with 0.2% (v/v) Triton X-100 for 10 min and blocked with PBS containing 5% (w/v) bovine serum albumin and 0.1% (v/v) Tween-20 for 30 min, the cells were incubated with rabbit anti-AC75 polyclonal antiserum (1:500) for 1 h followed by Alexa Fluor 594 goat anti-rabbit IgG (1:1000, Invitrogen) for 1 h in the dark. Subsequently, the cell nuclei were stained with Hoechst 33258 (Beyotime, Shanghai, China). All samples were observed with a 60× oil-immersion objective and a PerkinElmer UltraView VOX system.

### Quantitative Analysis of Viral Gene Transcription

Sf9 cells (1.0 × 10^6^ cells/plate) were transfected in triplicate with bAc*^*ac*75*F*54*S*^*-*ph* or bAc*^*ac*75^*^REP^-*ph* bacmid DNA and collected at 24 h post-transfection (p.t.). Total cellular RNA was isolated by using RNAiso Plus (Takara). The cDNA was then synthesized using an iScript cDNA synthesis kit (Takara). qPCR was performed with five pairs of specific viral gene primers (sequences are shown in Table 1) by a CFX96 real-time system (Bio-Rad) under the following conditions: denaturation at 95°C for 3 min followed by 40 cycles of 95°C for 10 s, 55°C for 30 s and 72°C for 20 s. Melting curve analysis was performed at the end of each PCR assay to test specificity (control). Host 18S rRNA was selected and used as the endogenous reference.

### Western Blot Analysis

Analysis of AC75 expression in cells infected with vAc*^*ac*75*F*54*S*^*-*ph* or vAc*^*ac*7^*^5REP^-*ph* over the course of infection was performed by western blot. Sf9 cells were infected in triplicate with vAc*^*ac*75*F*54*S*^*-*ph* or vAc*^*ac*7^*^5REP^-*ph* at an MOI of 5 and harvested at 18 and 24 h p.i. The cells were lysed on ice for 10 min in lysis buffer (Beyotime) and centrifuged in a microcentrifuge at 16,000 × *g* for 2 min. The lysates were subjected to western blot as described previously with some modifications (Xu et al., 2019). Anti-AC75 and anti-actin were used as primary antibodies. The signal was detected using a BeyoECL Plus Kit (Beyotime).

### Protein Stability Analysis

Protein stability was determined by using a previously described assay (Byers et al., 2016). Briefly, Sf9 cells were infected with vAc*^*ac*75*F*54*S*^*-*ph* or vAc*^*ac*75^*^REP^-*ph* at an MOI of five and were incubated with medium containing 400 μg/mL cycloheximide (CHX; Sigma) at 24 h p.i. The cells were harvested and lysed at the designated times, and the lysates were analyzed by western blot.

## Results

### *ac75* Is a Late Viral Gene

**FIGURE 1:**
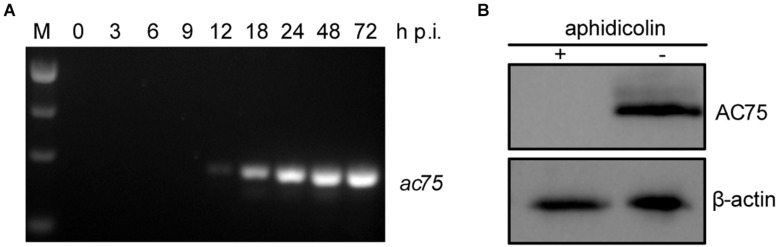
Analyses of the transcription and expression profile of *ac75* in infected cells. **(A)** Time course analysis of *ac75* transcription. Sf9 cells infected with AcMNPV were collected at the indicated time points. Total RNA was extracted and total cDNA was obtained by reverse transcription. *ac75* was amplified with specific primer pairs by PCR. **(B)** Western blot analysis of the expression of AC75 following aphidicolin treatment. AcMNPV infected cells were treated with 5 μg/mL aphidicolin (+) or DMSO (–) at 0 h p.i. The cells were lysed and subjected to western blot analysis with anti-AC75 and anti-actin as primary antibodies at 24 h p.i.

Transcriptomic analysis showed that three late transcription start sites are located upstream of the *ac75* translation initiation codon (Chen et al., 2013). Reverse transcription (RT)-PCR was performed in AcMNPV infected cells to confirm the temporal transcription patterns of *ac75*. The *ac75* transcripts were detected from 12 h p.i. and persisted up to 72 h p.i. (Figure 1A). Furthermore, western blot analysis indicated that aphidicolin inhibited AC75 expression (Figure 1B). These results confirmed that *ac75* was a late viral gene, implying its possible role during the late life cycle of AcMNPV.

### Phe-54 Is a Key Residue of AC75

**FIGURE 2:**
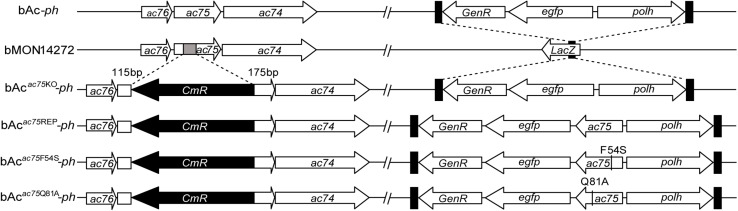
Schematic diagram of AcMNPV, *ac75* knockout, repaired and point mutation bacmids. The 112-bp fragment of *ac75* ORF in bMON14272 was replaced with a *CmR* cassette via homologous recombination to generate bAc*^*ac*75^*^KO^. bAc-*ph* and bAc*^*ac*75^*^KO^-*ph* were generated by inserting the *polh* and *egfp* genes into the *polh* locus of bMON14272 and bAc*^*ac*75^*^KO^, respectively. The *ac75*, *ac75*F54S or *ac75*Q81A controlled by the *ac75* native promoter with the *polh* and *egfp* genes were inserted into the *polh* locus of bAc*^*ac*75^*^KO^ to generate bAc*^*ac*75^*^REP^-*ph*, bAc*^*ac*75*F*54*S*^*-*ph* and bAc*^*ac*75*Q*81*A*^*-*ph*, respectively.

**FIGURE 3:**
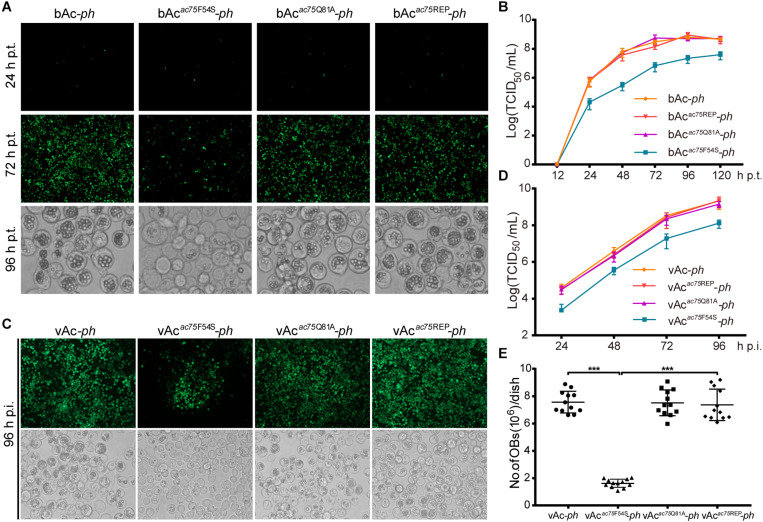
Analyses of viral replication and OB formation in the transfected/infected cells. **(A)** Fluorescence and light microscopy analyses of bacmid-transfected cells. Sf9 cells were transfected with bAc-*ph*, bAc*^*ac*75*F*54*S*^*-*ph*, bAc*^*ac*75*Q*81*A*^*-*ph* or bAc*^*ac*75^*^REP^-*ph* and imaged at 24, 72, and 96 h p.t. **(B)** Virus growth curves generated from bacmid-transfected cells. Sf9 cells were transfected with bAc-*ph*, bAc*^*ac*75*F*54*S*^*-*ph*, bAc*^*ac*75*Q*81*A*^*-*ph*, or bAc*^*ac*75^*^REP^-*ph*. The supernatants were harvested at the indicated time points, and virus titers were determined by the endpoint dilution assay. Each data point represents average titers from three separate transfections. Error bars represent standard deviations (SD). **(C)** Fluorescence and light microscopy analyses of virus-infected cells. Sf9 cells were infected with vAc-*ph*, vAc*^*ac*75*F*54*S*^*-*ph*, vAc*^*ac*75*Q*81*A*^*-*ph* or vAc*^*ac*75^*^REP^-*ph* (MOI = 0.5) at 96 h p.i. **(D)** Virus growth curves generated from virus-infected cells. Sf9 were infected with vAc-*ph*, vAc*^*ac*75*F*54*S*^*-*ph*, vAc*^*ac*75*Q*81*A*^*-*ph* or vAc*^*ac*75^*^REP^-*ph* at an MOI of 0.5. The supernatants were harvested at the designated times and determined by the endpoint dilution assay. Each data point represents average titers from three separate infections. Error bars represent SD. **(E)** Amount of OB production in each dish. Sf9 cells infected vAc-*ph*, vAc*^*ac*75*F*54*S*^*-*ph*, vAc*^*ac*75*Q*81*A*^*-*ph* or vAc*^*ac*75^*^REP^-*ph* (MOI = 10) were harvested at 96 h p.i. Total OBs of each dish were measured using a hemocytometer (*** indicates *P* < 0.001).

According to previous reports, *ac75* is an essential gene in the life cycle of the virus (Guo et al., 2017; Shi et al., 2017). Cells transfected with the *ac75*-null bacmid could not produce progeny BVs, which precluded analysis of the roles that AC75 plays throughout the life cycle of the virus. An InterProScan (Jones et al., 2014) and NCBI Conserved Domain Search (Marchler-Bauer et al., 2017) indicated that only a functionally unknown DUF1160 constitutes AC75, with no other annotated domains. To further investigate critical residues and functions of AC75, the sequences of AC75 homologs from 59 alphabaculoviruses and 14 betabaculoviruses were aligned by Clustal X-2.0 (Larkin et al., 2007). Residues Phe-54 and Gln-81 were found to be completely conserved among the homologs in the selected baculoviruses (Supplementary Figure 1). Based on this observation, we hypothesized that Phe-54 and Gln-81 from AC75 play key roles. Consequently, Phe-54 and Gln-81 point mutation bacmids (bAc*^*ac*75*F*54*S*^*-*ph* and bAc*^*ac*75*Q*81*A*^*-*ph*) were constructed (Figure 2). Sf9 cells were transfected with bAc*^*ac*75*F*54*S*^*-*ph*, bAc*^*ac*75*Q*81*A*^*-*ph*, bAc*^*ac*75^*^REP^-*ph* or bAc-*ph* and monitored by fluorescent microscopy. There were no significant differences in the number of fluorescent cells among bacmids at 24 h p.t. (Figure 3A, upper panel), indicating relatively equal frequencies of transfection of these bacmids. By 72 h p.t., most of the bAc*^*ac*75*Q*81*A*^*-*ph*-, bAc*^*ac*75^*^REP^-*ph*- or bAc-*ph*-transfected cells exhibited fluorescence, whereas only a small proportion of bAc*^*ac*75*F*54*S*^*-*ph*-transfected cells showed fluorescence (Figure 3A, middle panel). In addition, light microscopy images revealed that there were fewer intracellular OBs in bAc*^*ac*75*F*54*S*^*-*ph*-transfected cells when compared with cells transfected with the other bacmids (Figure 3A, lower panel), suggesting that the AC75-F54S mutation may also affect OB production. Furthermore, virus growth curve analysis showed that bAc*^*ac*75*Q*81*A*^*-*ph*, bAc*^*ac*75^*^REP^-*ph* and bAc-*ph* had comparable growth kinetics, whereas bAc*^*ac*75*F*54*S*^*-*ph* produced fewer progeny BVs from 24 to 120 h p.t. (Figure 3B). Taken together, these results revealed that Phe-54 is a key residue of AC75, and the AC75-F54S mutation may affect progeny BV production and OB formation.

To further confirm the results obtained from bacmid transfection, Sf9 cells were infected with vAc-*ph*, vAc*^*ac*75*F*54*S*^*-*ph*, vAc*^*ac*75*Q*81*A*^*-*ph* or vAc*^*ac*75^*^REP^-*ph* at an MOI of 0.5, and virus growth curve analysis was performed. At 96 h p.i., the number of fluorescent cells infected with mutant virus vAc*^*ac*75*F*54*S*^*-*ph* was less than those infected with vAc-*ph*, vAc*^*ac*75*Q*81*A*^*-*ph* or vAc*^*ac*75^*^REP^-*ph* (Figure 3C, upper panel), and the number of OBs produced by vAc*^*ac*75*F*54*S*^*-*ph*-infected cells was also less when compared with those cells infected with vAc-*ph*, vAc*^*ac*75*Q*81*A*^*-*ph* or vAc*^*ac*75^*^REP^-*ph* (Figure 3C, lower panel). The BV production of the vAc*^*ac*75*F*54*S*^*-*ph* virus was reduced by 10-fold when compared with those of vAc-*ph*, vAc*^*ac*75*Q*81*A*^*-*ph* or vAc*^*ac*75^*^REP^-*ph* from 24 to 96 h p.i. (Figure 3D). The number of OBs in cells infected with vAc-*ph*, vAc*^*ac*75*F*54*S*^*-*ph*, vAc*^*ac*75*Q*81*A*^*-*ph*, or vAc*^*ac*75^*^REP^-*ph* at an MOI of 10 were counted. The OBs of vAc*^*ac*75*F*54*S*^*-*ph-*infected cells from each dish were reduced by approximately eight-fold when compared with those cells infected with vAc-*ph*, vAc*^*ac*75*Q*81*A*^*-*ph* or vAc*^*ac*75^*^REP^-*ph* at 96 h p.i. (*P* < 0.001) (Figure 3E). These observations validated that the AC75-F54S mutation affected BV production and OB formation.

### The AC75-F54S Mutation Affected Polyhedrin Assembly and ODV Embedding

**FIGURE 4:**
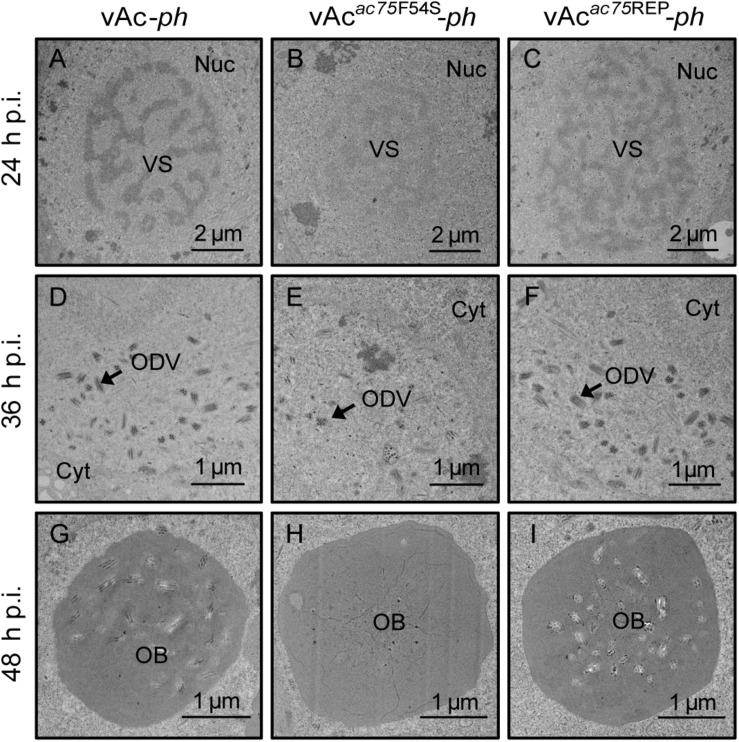
TEM analysis of cells infected with vAc-*ph*, vAc*^*ac*75*F*54*S*^*-*ph*, or vAc*^*ac*75^*^REP^-*ph*. Sf9 cells were infected with vAc-*ph*, vAc*^*ac*75*F*54*S*^*-*ph* or vAc*^*ac*75^*^REP^-*ph*. The cells were collected at 24, 36, and 48 h p.i., and prepared for TEM. **(A–C)** The enlarged nucleus (Nuc) and virogenic stroma (VS) in vAc-*ph*, vAc*^*ac*75*F*54*S*^*-*ph* or vAc*^*ac*75^*^REP^-*ph*-infected cells at 24 h p.i. **(D–F)** The normal ODVs in the nucleus ring zone of cells infected with vAc-*ph*, vAc*^*ac*75*F*54*S*^*-*ph* or vAc*^*ac*75^*^REP^-*ph* at 36 h p.i. Cyt, cytoplasm. **(H)** The OB with a few ODVs in vAc*^*ac*75*F*54*S*^*-*ph*-cells at 48 h p.i. **(G,I)** Normal OBs in vAc-*ph* or vAc*^*ac*75^*^REP^-*ph*-infected cells at 48 h p.i.

Transmission electron microscopy was performed to further determine the effects of the AC75-F54S mutation on intranuclear structures. At 24 and 36 h p.i., vAc*^*ac*75*F*54*S*^*-*ph*-infected cells exhibited typical baculovirus infection symptoms, including a typical VS and abundant rod-shaped nucleocapsids (Figure 4B), and ODVs with nucleocapsids (Figure 4E). As expected, vAc-*ph*- and vAc*^*ac*75^*^REP^-*ph*-infected cells also showed a typical VS and normal rod-shaped nucleocapsids (Figures 4A,C), and numerous ODVs (Figures 4D,F) at 24 and 36 h p.i. However, the OBs of vAc*^*ac*75*F*54*S*^*-*ph*-infected cells contained fewer envelope virions (Figure 4H) when compared with those of vAc-*ph* and vAc*^*ac*75^*^REP^-*ph*-infected cells (Figures 4G,I) at 48 h p.i., indicating that the AC75-F54S mutation impaired OB occlusion ODVs.

**FIGURE 5:**
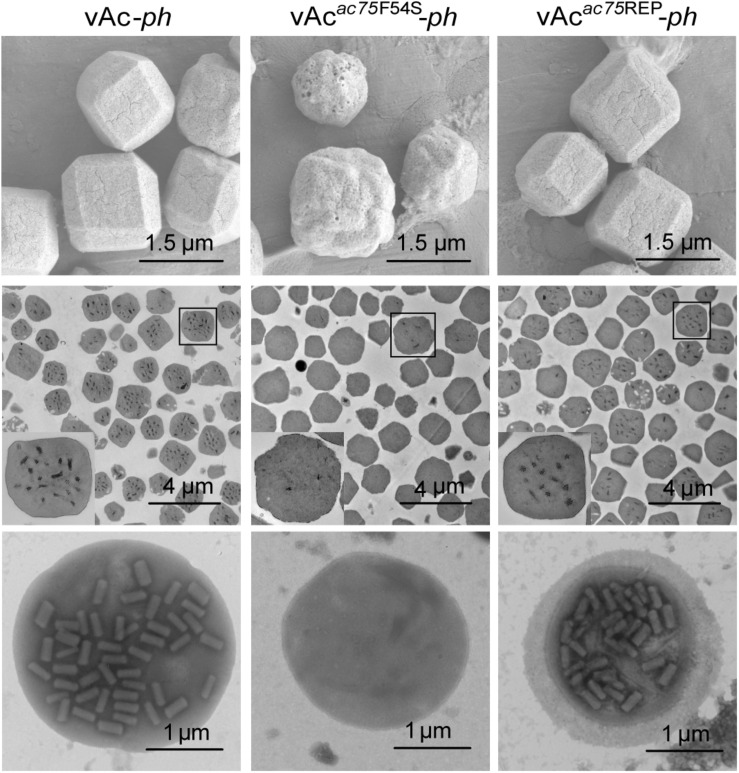
SEM, TEM and negative staining analyses of OBs. The OBs were purified from *S. exigua* larvae infected with vAc-*ph*, vAc*^*ac*75*F*54*S*^*-*ph* or vAc*^*ac*75^*^REP^-*ph*. (Upper panel) SEM analyses of OBs. The images show the external morphology of OBs. (Middle panel) TEM analyses of OBs. The ultrathin sections enabled visualization of the internal structure of OBs. Boxed regions of the OB are magnified and shown in the inset. (Lower panel) Negative staining analyses of OBs.

To further investigate the effects of the AC75-F54S mutation on OB morphogenesis, we performed SEM and TEM on OBs purified from vAc-*ph*-, vAc*^*ac*75*F*54*S*^*-*ph*- or vAc*^*ac*75^*^REP^-*ph*- infected *S. exigua* cadavers. SEM analysis showed that the OBs from vAc*^*ac*75*F*54*S*^*-*ph*-infected larvae had ragged surfaces and irregular shapes, whereas the OBs of vAc-*ph* or vAc*^*ac*75^*^REP^-*ph*-infected larvae had smooth surfaces and sharp edges (Figure 5, upper panel). TEM images showed that only a few OBs of vAc*^*ac*75*F*54*S*^*-*ph* contained ODVs, whereas the majority of OBs of vAc-*ph* and vAc*^*ac*75^*^REP^-*ph* occluded normal ODVs with multi-nucleocapsids (Figure 5, middle panel). Furthermore, twenty visual fields were randomly selected and analyzed, the proportion of vAc*^*ac*75*F*54*S*^*-*ph* OBs with ODVs were only 7.8%. In addition, OBs were dissolved, negative stained and examined by TEM. Many OBs of vAc*^*ac*75*F*54*S*^*-*ph* were empty (Figure 5, lower panel), which is consistent with the observations of thin sections of OBs, whereas OBs of vAc-*ph* and vAc*^*ac*75^*^REP^-*ph* contained normal single-nucleocapsids and multi-nucleocapsids (Figure 5, lower panel). According to these results, the AC75-F54S mutation caused aberrant polyhedrin assembly and ODV embedding during OB formation.

### The AC75-F54S Mutation Did Not Affect Polyhedrin Expression and Localization

**FIGURE 6:**
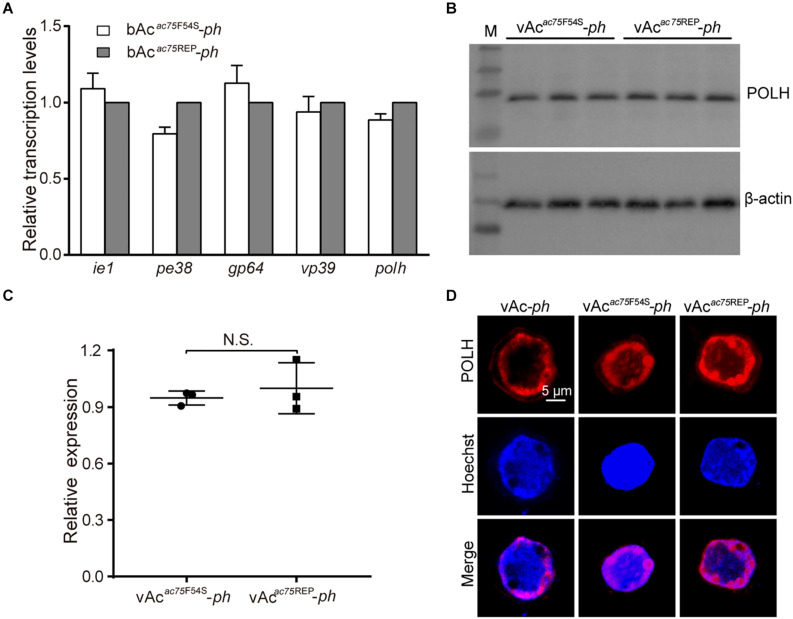
Transcription, expression and localization analyses of *polyhedrin*. **(A)** RT-qPCR analysis. Total RNA was extracted from Sf9 cells transfected with either bAc*^*ac*75*F*54*S*^*-*ph* or bAc*^*ac*75^*^REP^-*ph* at 24 h p.t. Transcription of selected viral genes was measured by RT-qPCR. The transcript level of each gene was normalized by 18S rRNA of the same sample. The values represent the averages from three independent assays and error bars represent SD. **(B)** Western blot analysis. Sf9 cells infected with vAc*^*ac*75*F*54*S*^*-*ph* or vAc*^*ac*75^*^REP^-*ph* at an MOI of five were collected and lysed at 24 h p.i. The lysates were subjected to western blot analysis using anti-POLH and anti-actin (control) as primary antibodies. **(C)** Relative quantification of the expression levels of POLH by densitometry analysis of western blot. Each dot represents the expression level in one independent experiment relative to the mean for POLH in vAc-*ph*-infected cells at 24 h p.i. The values represent the averages from three independent assays and error bars represent SD. N.S. indicates no significance, *P* > 0.05. **(D)** Immunofluorescence analysis. Sf9 cells infected with vAc-*ph*, vAc*^*ac*75*F*54*S*^*-*ph* or vAc*^*ac*75^*^REP^-*ph* at an MOI of 5 were fixed at 24 h p.i. and stained. The POLH are in red and nuclei are stained blue.

According to previous reports, *ac75* is essential in the life cycle of the virus but deleting *ac75* did not affect viral genome replication (Guo et al., 2017; Shi et al., 2017). To investigate whether the AC75-F54S mutation affected transcription of viral genes, five viral genes, including two early genes (*ie1* and *pe38*), one early-late gene (*gp64*) and two late genes (*vp39* and *polh*), were selected and analyzed. The transcript levels of the selected genes were determined by RT-qPCR with the corresponding primers (sequences are shown in Table 1). As shown in Figure 6A, no significant differences in transcript levels of all selected genes were observed between bAc*^*ac*75*F*54*S*^*-*ph*- and bAc*^*ac*75^*^REP^-*ph-*transfected cells (*P* > 0.05). The results showed that the AC75-F54S mutation did not affect early or late viral gene transcription.

Western blot analysis was performed to determine whether the AC75-F54S mutation affected expression of the polyhedrin protein because the AC75-F54S mutation was found to impair OB formation. At 24 h p.i., there were no difference among the polyhedrin expression levels of vAc*^*ac*75*F*54*S*^*-*ph*- and vAc*^*ac*75^*^REP^-*ph*-infected cells (Figures 6B,C), indicating that the AC75-F54S mutation also did not affect polyhedrin expression. Furthermore, an immunofluorescence assay was performed to investigate the effect of the AC75-F54S mutation on polyhedrin localization. As shown in Figure 6D, polyhedrins were distributed predominantly in the nucleus, exhibiting a ring pattern in the ring zone, with a weak distribution in the cytoplasm of vAc-*ph*, vAc*^*ac*75*F*54*S*^*-*ph*- and vAc*^*ac*75^*^REP^-*ph*-infected cells at 24 h p.i. The results indicated that the AC75-F54S mutation did not affect the localization of the polyhedrin. Taken together, these data suggested that the AC75-F54S mutation might impair OB formation by affecting polyhedrin assembly.

### The AC75-F54S Mutation Caused a Decreased in the Amount of AC75

**FIGURE 7:**
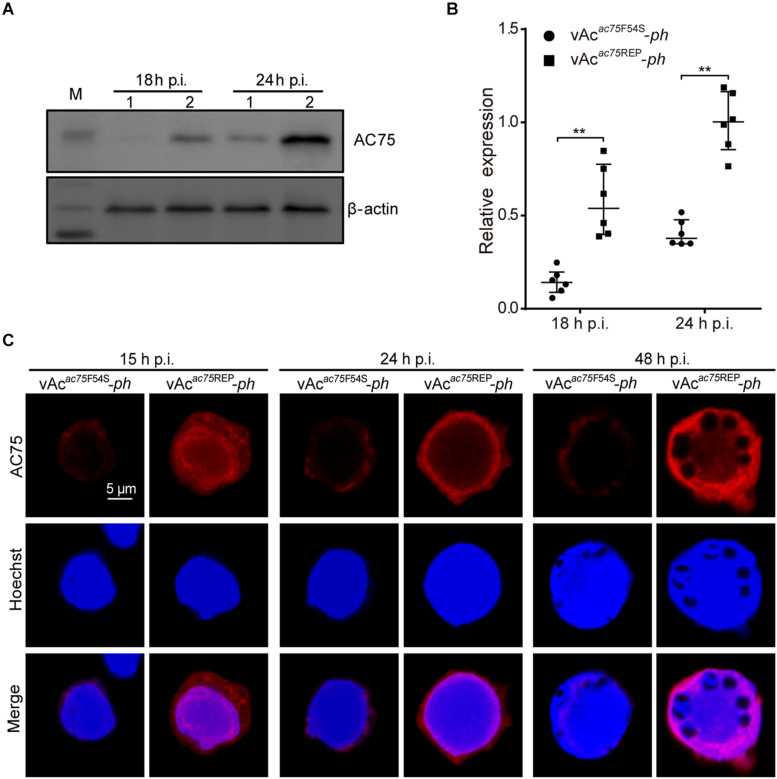
Expression and localization of AC75 in vAc*^*ac*75*F*54*S*^*-*ph*- or vAc*^*ac*75^*^REP^-*ph*-infected cells. **(A)** Western blot analysis. Sf9 cells infected with vAc*^*ac*75*F*54*S*^*-*ph* or vAc*^*ac*75^*^REP^-*ph* at an MOI of 5 were harvested and lysed at 18 and 24 h p.i. The lysates were subjected to western blot using anti-AC75 and anti-actin (control) as primary antibodies. Lane 1, vAc*^*ac*75*F*54*S*^*-*ph*; Lane 2, vAc*^*ac*75^*^REP^-*ph*. **(B)** Relative quantification of the expression levels of AC75 by densitometry analysis of western blot. Each dot represents the expression level in one independent experiment relative to the mean for AC75 in vAc*^*ac*75^*^REP^-*ph*-infected cells at 24 h p.i. The values represent the averages from six independent assays and error bars represent SD. ** indicates *P* < 0.01. **(C)** Immunofluorescence analysis of localization and expression of AC75. Sf9 cells infected with vAc*^*ac*75*F*54*S*^*-*ph* or vAc*^*ac*75^*^REP^-*ph* at an MOI of 5 were fixed at the indicated time points. The cells were incubated with an anti-AC75 antibody (red). The nuclei were stained with Hoechst 33258 (blue).

To further investigate the nature of the defect caused by the AC75-F54S mutation, the relative protein expression levels of AC75 in vAc*^*ac*75*F*54*S*^*-*ph*- or vAc*^*ac*75^*^REP^-*ph*-infected cells were compared over the course of infection by western blot analysis. As shown in Figures 7A, AC75 was detected in both vAc*^*ac*75*F*54*S*^*-*ph*- and vAc*^*ac*75^*^REP^-*ph*-infected cells at 18 and 24 h p.i.; however, the protein band representing AC75 in vAc*^*ac*75*F*54*S*^*-*ph*-infected cells was much weaker in intensity when compared with that of vAc*^*ac*75^*^REP^-*ph*-infected cells. The protein expression levels were further examined by standardization against actin expression and densitometry analysis. The relative AC75 protein expression in vAc*^*ac*75^*^REP^-*ph-*infected cells was 0.58 ± 0.19 and 1.00 ± 0.16 at 18 and 24 h p.i., respectively (Figure 7B). In contrast, the relative AC75 protein expression in vAc*^*ac*75*F*54*S*^*-*ph*-cells was 0.15 ± 0.07 and 0.41 ± 0.07 at 18 and 24 h p.i., respectively (Figure 7B). Immunoelectron microscopy analysis was performed with thin sections generated from cells infected with vAc-*ph*, vAc*^*ac*75*F*54*S*^*-*ph* or vAc*^*ac*75^*^REP^-*ph*, and fixed at 48 h p.i. In agreement with the above results, the number of colloidal-gold-labeled AC75 in the vAc*^*ac*75*F*54*S*^*-*ph*-infected cells was remarkably lower when compared with that of vAc-*ph*- or vAc*^*ac*75^*^REP^-*ph*-infected cells (Supplementary Figure 2).

Furthermore, immunofluorescence assays were performed to investigate the effect of the AC75-F54S mutation on the localization and abundance of AC75. At 15 h p.i., the AC75 signal was distributed uniformly throughout cells infected with vAc*^*ac*75*F*54*S*^*-*ph* or vAc*^*ac*75^*^REP^-*ph* (Figure 7C). By 24 and 48 h p.i., AC75 was localized predominantly within the nucleus, particularly around the nuclear rim and became condensed to the intranuclear ring zone in vAc*^*ac*75*F*54*S*^*-*ph*- and vAc*^*ac*75^*^REP^-*ph*-infected cells (Figure 7C). Thus, the localization of AC75 was similar in vAc*^*ac*75*F*54*S*^*-*ph*- and vAc*^*ac*75^*^REP^-*ph*-infected cells. In contrast, the AC75 signal in vAc*^*ac*75*F*54*S*^*-*ph*-infected cells was much weaker when compared with those in vAc*^*ac*75^*^REP^-*ph*- and vAc-*ph*-infected cells at all-time points (Figure 7C). The localization and abundance of AC75 in vAc-*ph*-infected cells were comparable with those of vAc*^*ac*75^*^REP^-*ph* (Supplementary Figure 3). Taken together, these data demonstrated that the AC75-F54S mutation did not affect localization of AC75 in infected cells but caused a reduction in the amount of AC75.

### The AC75-F54S Mutation Affected the Stability of AC75

**FIGURE 8:**
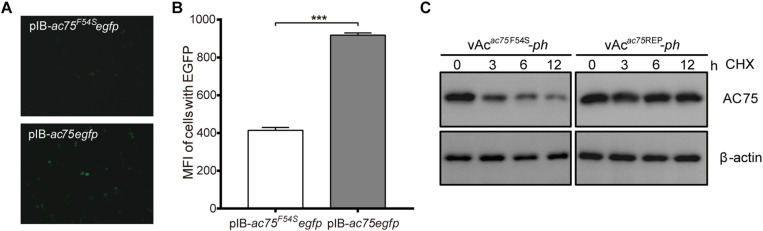
Stability analysis of AC75. **(A)** Fluorescence analyses. Sf9 cells transfected with pIB-*ac75*^*F*54*S*^*egfp* or pIB-*ac75egfp* plasmids were imaged at 48 h p.t. **(B)** Flow cytometry analyses. The mean fluorescence intensity of cells expressing the EGFP gene were analyzed by flow cytometry analysis. The values represent averages from three independent assays and error bars represent SD (*** indicates *P* < 0.001). **(C)** Western blot analysis. Sf9 cells infected with vAc*^*ac*75*F*54*S*^*-*ph* or vAc*^*ac*75^*^REP^-*ph* in triplicate at an MOI of 5 were treated with CHX at 24 h p.i. Lysates prepared at the indicated times thereafter were subjected to immunoblot analysis by using anti-AC75 and anti-actin antibodies.

The decrease in the amount of AC75 is probably because translation or stability of AC75 is affected by the AC75-F54S mutation. We initially examined and compared the expression of AC75 in cells transfected with pIB-*ac75^*F*54*S*^egfp* or pIB-*ac75egfp* plasmids to determine whether the translation or the stability of AC75 was affected. The AC75 signal of cells transfected with pIB-*ac75^*F*54*S*^egfp* was much weaker than that of pIB-*ac75egfp* with the same exposure time at 48 h p.t. (Figure 8A). Subsequently, we collected the cells and analyzed the mean fluorescence intensity of cells with EGFP by flow cytometry. As shown in Figure 8B, the mean fluorescence intensity of cells transfected with pIB-*ac75^*F*54*S*^egfp* was significantly lower than that of pIB-*ac75egfp* (*P* < 0.001). These results indicated that the AC75-F54S mutation might impair AC75 stability.

To confirm the above results, a cycloheximide (CHX) treated assay was used to evaluate the relative stability of AC75. Briefly, Sf9 cells infected with vAc*^*ac*75*F*54*S*^*-*ph* or vAc*^*ac*75^*^REP^-*ph* at an MOI of five were treated with 400 μg/mL CHX to block protein synthesis at 24 h p.i. The cells were collected and lysed at 0, 3, 6 and 12 h after CHX treatment, and the lysates were analyzed by western blot. The AC75 protein level in vAc*^*ac*75*F*54*S*^*-*ph-*infected cells was reduced significantly from 0 to 12 h (Figure 8C). In contrast, there were no obvious changes in AC75 protein levels in vAc*^*ac*75^*^REP^-*ph-*infected cells following CHX treatment (Figure 8C). Accordingly, these data are consistent with the flow cytometry results presented above. Taken together, these data suggest that the AC75-F54S mutation affects the relative stability of AC75 in infected cells.

## Discussion

*ac75* is a highly conserved gene in all sequenced baculovirus genomes except CuniNPV. According to previous reports, *ac75* is essential for nuclear egress and intranuclear microvesicle formation (Guo et al., 2017; Shi et al., 2017). Deletion of *ac75* resulted in a complete loss of viral growth in cultured cells, which precluded the investigation of AC75 function during late infection. In this study, we identified that Phe-54 was an important residue of AC75, and validated the important role of *ac75* in polyhedrin assembly and ODV embedding during OB formation.

Previous transcriptome analysis identified three typical late promoters located at 282, 93, and 14 nt upstream of the *ac75* start codon in *Trichoplusia ni* (*T. ni*) cells infected with AcMNPV (Chen et al., 2013). In this study, the transcription pattern revealed that *ac75* was transcribed from 12 h p.i. and this transcription persisted up to 72 h p.i. These observations are in accord with previous results showing that *ac75* was expressed at relatively high levels from 18 to 72 h p.i. in the AcMNPV-infected midgut of *T. ni* (Shrestha et al., 2018). In addition, western blot analysis revealed that AC75 was expressed during the late infection phase. Thus, *ac75* may play a role during the late stage of infection. Shi et al. (2017) and Guo et al. (2017) found that *ac75* was essential for nuclear egress and intranuclear microvesicle formation. However, the possible functions of *ac75* in other processes during the late infection phase have not been investigated in detail.

Sequence alignment of AC75 homologs showed that Phe-54 and Gln-81 were completely conserved among the selected homologs. Phe is a hydrophobic amino acid with an aromatic side chain, whereas Gln has a polar, non-charged side chain. In this study, Phe-54 was mutated to glycine (F54G), alanine (F54A), or serine (F54S). These mutant viruses were transfected/infected into Sf9 cells, revealing that the AC75-F54S mutation produced 10-fold fewer progeny BVs than the wild-type virus, whereas the AC75-F54G mutation exhibited a modest two-fold reduction in viral titer when compared with that of the wild-type virus. The AC75-F54A mutation did not affect BV production (data not shown). Mutation of Gln-81 to glycine (Q81G), alanine (Q81A) or glutamic acid (Q81E) did not affect viral propagation (data not shown), indicating that residue Gln-81 may not be important for the functions of AC75. Thus, Phe-54 may be an important residue of AC75, and the AC75-F54S mutant virus was chosen for subsequent research.

In this study, novel functions of AC75 were investigated through characterization of the AC75-F54S partial loss-of-function mutant virus during the late infection phase. Electron microscopy revealed that the OBs of vAc*^*ac*75*F*54*S*^*-*ph* had a ragged surface and contained markedly low numbers of ODVs and most OBs of the vAc*^*ac*75*F*54*S*^*-*ph* were empty. In addition, the AC75-F54S mutation did not affect expression and localization of the polyhedrin. Thus, *ac75* may play a key role in the assembly of the polyhedrin and the embedding of ODVs during OB morphogenesis. The process of OB occlusion requires a complex integration of events, including envelopment of ODVs, polyhedrin assembly and incorporation of ODVs. The mature ODVs associate with dense concentrations of the polyhedral protein, which subsequently crystallizes around one or many ODVs to form OBs. The mechanisms that control crystallization at the ODV-polyhedrin interface are unresolved and the viral proteins required for this process are unknown. The surface layer (calyx or polyhedral envelope) of mature OBs is composed of carbohydrates and PEP (Whitt and Manning, 1988). The formation of this layer structure requires a functional P10 and is associated with nuclear fibrillar structures (Carpentier et al., 2008). The OBs of a *pep* or *p10* mutant virus are irregular and fragile with no outer layer (Vlak et al., 1988; Li et al., 2015). According to recent reports, the OB morphology was also abnormal in a P33 point mutation and a VP91 truncated mutation (Kuang et al., 2017; Zhou et al., 2019), suggesting that both P33 and VP91 are involved in OBs morphogenesis, which involves assembly of the polyhedrin and the embedding of ODVs. Therefore, *ac75* is the third gene identified to be involved with the assembly of the polyhedrin and embedding of ODVs. Further studies examining genes associated with OB formation will aid our understanding of this molecular mechanism.

Further investigation revealed that the AC75-F54S mutation reduced the amount of AC75, but did not affect localization in infected cells. We hypothesized that the lower amount of AC75 in vAc*^*ac*75*F*54*S*^*-*ph*-infected cells affected the efficiency of nuclear egress and therefore resulted in lower amounts of BV production. Flow cytometry and western blot analyses revealed that the lower levels of AC75-F54S might be a result of impaired stability caused by AC75-F54S mutation. The loss of persistence of AC75 may be caused by structural disorder. Thus, the AC75-F54S mutation may have caused mis-folding of AC75, which further affected its proper functions in the AcMNPV life cycle. Moreover, incorrectly folded AC75 may be degraded by the 20S proteasome (Asher et al., 2006; Tompa et al., 2008). Further analysis is required to determine the exact functional role of Phe-54 in AC75 activity in AcMNPV-infected cells.

In conclusion, we revealed that the residue Phe-54 in AC75 is important for the proper folding of this protein, and AC75 is involved in polyhedrin assembly and ODV embedding during OB formation. Thus, *ac75* is a versatile gene associated with nuclear egress of nucleocapsids and OB morphogenesis. Moreover, our results provide evidence for a pivotal role of *ac75* in BV production and OB formation, and further studies of AC75 are required to elucidate the relation between BV formation and OB morphogenesis. These data may help uncover the mechanism of OB morphogenesis of baculoviruses.

## Data Availability Statement

The datasets presented in this study can be found in online repositories. The names of the repository/repositories and accession number(s) can be found in the article/Supplementary Material.

## Ethics Statement

The animal study was reviewed and approved by the Institutional Review Board, Wuhan Institute of Virology, Chinese Academy of Sciences.

## Author Contributions

XC, XS, and JH designed the experiments and wrote the manuscript. XC, XY, and CL carried out the experiments and analyzed the data. XC, JY, XS, and JH checked and finalized the manuscript. All authors contributed to manuscript revision, read, and approved the submitted version.

## Conflict of Interest

The authors declare that the research was conducted in the absence of any commercial or financial relationships that could be construed as a potential conflict of interest.

## Footnotes

1 https://imagej.nih.gov/ij/

## Supplementary Material

Supplementary Figure 1 Sequence alignment of the AC75 homologs from 54 alphabaculoviruses and 12 betabaculoviruses. Amino acid sequences were aligned using Clustal X-2.0. Black shading denotes identical conserved residues, whereas gray shading indicates less conserved sites. Residues Phe-54 and Gln-81 were absolutely conserved among the selected homologs. The GenBank accession numbers of the amino acid sequences are presented in Supplementary Table 1.

Supplementary Figure 2 Immunoelectron microscopy analysis. Sf9 cells were infected with vAc-*ph*, vAc*^*ac75F54S*^*-*ph*, or vAc*^*ac75*^*^REP^-*ph* at an MOI of five and harvested at 48 h p.i. Ultrathin sections were probed with the anti-AC75 antibody as the primary antibody and goat anti-rabbit IgG coated with gold particles (10 nm) as the secondary antibody.

Supplementary Figure 3 Immunofluorescence analysis of localization and expression of AC75 in vAc-*ph*-infected cells. Sf9 cells were infected with vAc-*ph* (MOI = 5) and fixed at the indicated time points. The cells were incubated with an anti-AC75 antibody (red). The nuclei were stained with Hoechst 33258 (blue).

Supplementary Table 1 The GenBank accession numbers of the amino acid sequences of AC75 homolog proteins selected in this study.
