# Supplementary figures and images for: A Conserved Phenylalanine Residue of Autographa Californica Multiple Nucleopolyhedrovirus AC75 Protein Is Required for Occlusion Body Formation
Source: Front Microbiol. 2021 Apr 8;12:663506. doi: 10.3389/fmicb.2021.663506 (PMC8060461; doi:10.3389/fmicb.2021.663506)

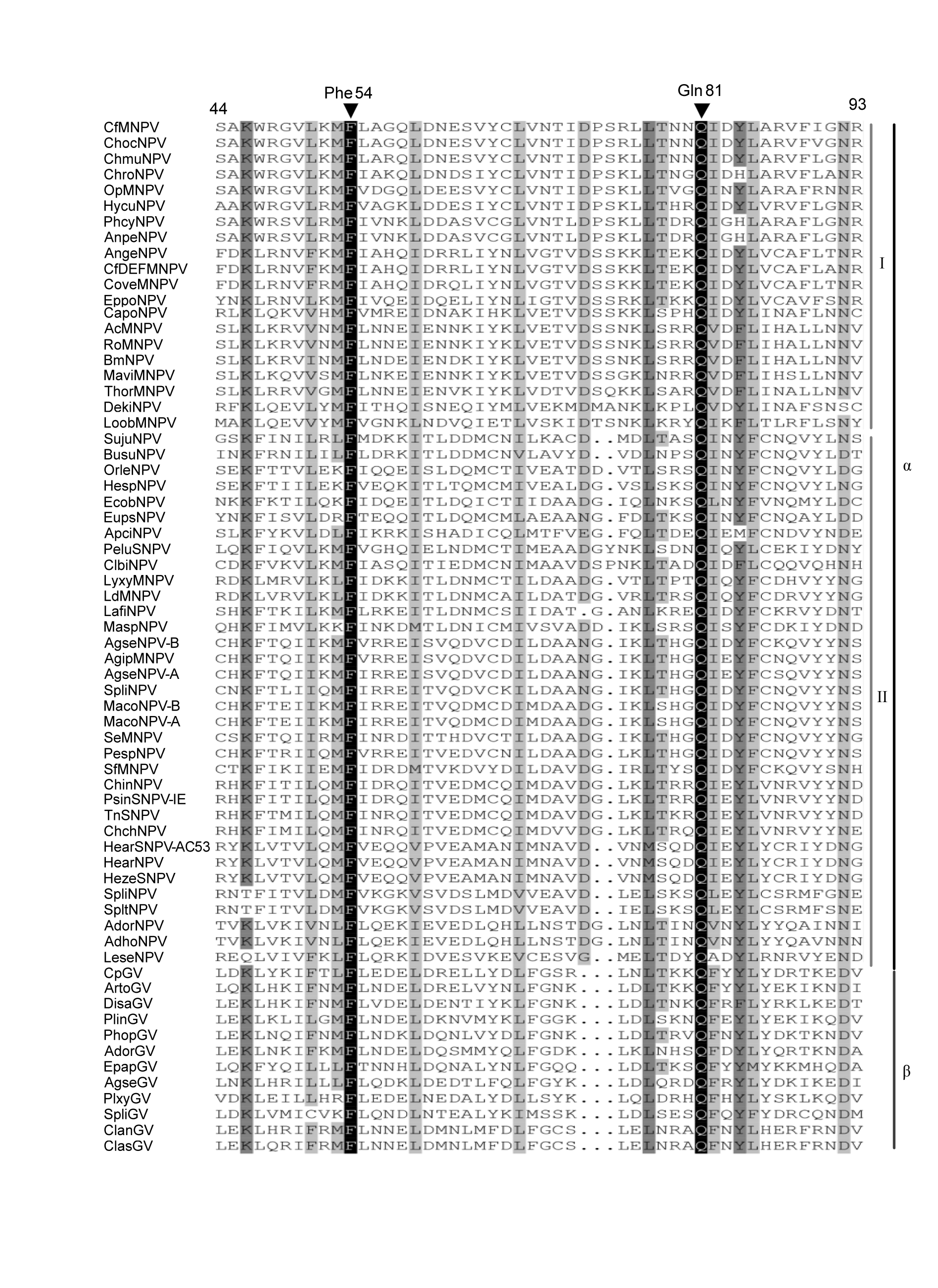

Supplement: Supplementary Figure 1 — Sequence alignment of the AC75 homologs from 54 alphabaculoviruses and 12 betabaculoviruses. Amino acid sequences were aligned using Clustal X-2.0. Black shading denotes identical conserved residues, whereas gray shading indicates less conserved sites. Residues Phe-54 and Gln-81 were absolutely conserved among the selected homologs. The GenBank accession numbers of the amino acid sequences are presented in Supplementary Table 1. [file Image_1.TIF]

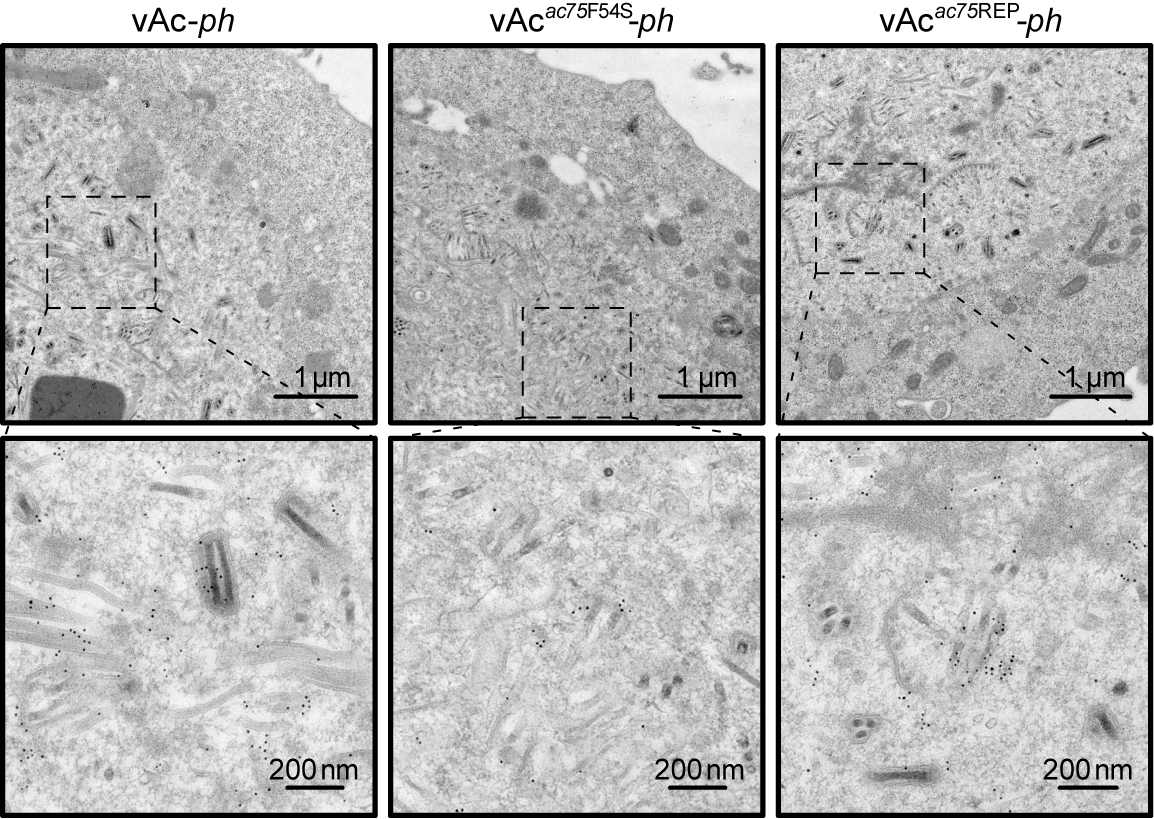

Supplement: Supplementary Figure 2 — Immunoelectron microscopy analysis. Sf9 cells were infected with vAc-ph, vAcac75F54S-ph, or vAcac75REP-ph at an MOI of five and harvested at 48 h p.i. Ultrathin sections were probed with the anti-AC75 antibody as the primary antibody and goat anti-rabbit IgG coated with gold particles (10 nm) as the secondary antibody. [file Image_2.TIF]

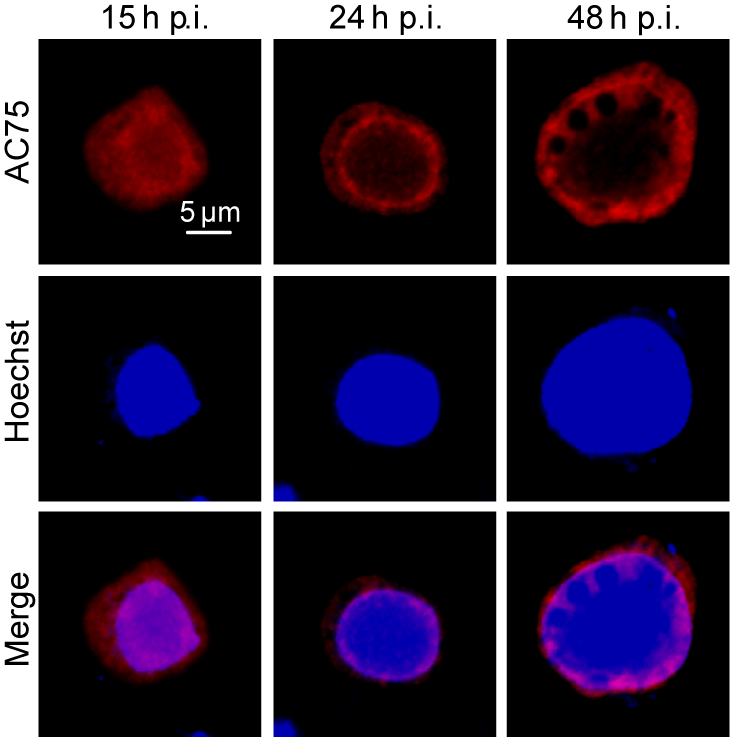

Supplement: Supplementary Figure 3 — Immunofluorescence analysis of localization and expression of AC75 in vAc-ph-infected cells. Sf9 cells were infected with vAc-ph (MOI = 5) and fixed at the indicated time points. The cells were incubated with an anti-AC75 antibody (red). The nuclei were stained with Hoechst 33258 (blue). [file Image_3.TIF]
